# Senescence marker protein 30 (SMP30) serves as a potential prognostic indicator in hepatocellular carcinoma

**DOI:** 10.1038/srep39376

**Published:** 2016-12-19

**Authors:** Zhijing Mo, Shunxin Zheng, Zhilue Lv, Yuan Zhuang, Xiuwan Lan, Feng Wang, Xiaoling Lu, Yongxiang Zhao, Sufang Zhou

**Affiliations:** 1 Department of Biochemistry and Molecular Biology, Guangxi Medical University, Nanning 530021, China; 2 Guangxi Key Laboratory of Biological Targeting Diagnosis and Therapy Research, Guangxi Medical University, Nanning 530021, China

## Abstract

Senescence marker protein 30 (SMP30) has been identified as a tumor-related molecule of hepatocellular carcinoma (HCC). Its clinical significance and underlying mechanisms in HCC tissues, however, remain largely unexplored. We have demonstrated a preferentially expressed SMP30 in normal liver using a tissue microarray. By employing real-time quantitative PCR, two tissue microarrays and Oncomine database analysis, we have also shown that the SMP30 in HCC tissues has significantly reduced when compared with that in paired adjacent non-tumor tissues (*P* = 0.0037). The reduced expression of SMP30 is very noticeably related to larger tumor size (*P* = 0.012), enhanced TNM (*P* = 0.009) and worse survival (*P* < 0.0001) in HCC patients. The analyses using Cox regression have indicated that the decreased SMP30 expression is an independent risk to the reduced overall survival rate of HCC patients (*P* = 0.001), and the down-regulation of SMP30 in HCC might be mediated by DNA methylation. Moreover, genes co-expressed with SMP30 may affect the prognosis through apoptotic process, biological adhesion and blood coagulation by PANTHER analyses. Our studies have indicated that the SMP30 may serve as a candidate of HCC clinical prognostic marker and a potential therapeutic target.

It is well known that majority (70–90%) of primary hepatic carcinoma diagnosed worldwide is hepatocellular carcinoma (HCC)^1^ and that the highest liver cancer incidence is found in East and South-East Asia, followed by Northern and Western Africa^2^. Although advances in medicines have raced ahead in the treatment of HCC, its long-term survival rate is limited by the high rate of recurrence and metastasis after surgery^3^. It is unclear, however, how the molecular mechanism contributes to recurrence and metastasis of HCC. Thus, it is necessary to investigate and develop novel diagnosis, prognosis and individualized medication biomarkers to improve clinical outcomes in patients with HCC.

Senescence marker protein 30 (SMP30) is a calcium binding protein. We have previously used SEREX^4^ approach and identified SMP30 from Guangxi HCC cDNA expression library. Studies have indicated that autoantibody to SMP30 could be one of serum diagnostic indices for HCC^5^. Inhibitions of SMP30 in the HCC cell line Hep G2 have been shown to promote the cell proliferation, cell cycle, apoptosis and invasion^6^. The correlations between SMP30 expression and prognostic significance in HCC, however, have not been reported.

The purpose of this research work is to investigate the SMP30 expression status in human normal tissues, in HCC tissues and in their adjacent non-tumor tissues. The SMP30 expression and its correlations with clinical characteristics including overall survival (OS) rate have been assessed. We have also discussed the main mechanism that account for SMP30 losses in HCC patients and performed a biologic process analysis of genes co-expressed with SMP30.

## Results

### The SMP30 expression in normal tissues and organs

**Figure 1:**
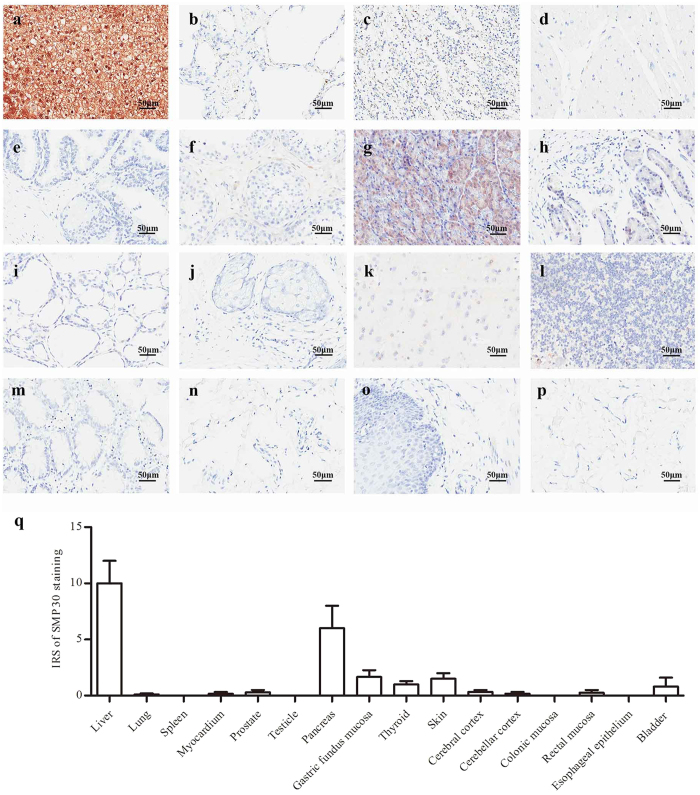
Showing that SMP30 expression is high in normal liver tissue. Immunohistochemical representative images of SMP30 expression in (**a**) Liver. (**b**) Lung. (**c**) Spleen. (**d**) Myocardium. (**e**) Prostate. (**f**) Testicle. (**g**) Pancreas. (**h**) Gastric fundus mucosa. (**i**) Thyroid. (**j**) Skin. (**k**) Cerebral cortex. (**l**) Cerebellar cortex. (**m**) Colonic mucosa. (**n**) Rectal mucosa. (**o**) Esophageal epithelium. (**p**) Bladder epithelium. The original magnification of IHC staining is 200 × . (**q**) Immunoreactivity score (IRS) of SMP30 staining was available from 16 human normal tissues and organs. The data represent the mean ± SD. *P* < 0.05.

To determine whether SMP30 expression is different in various human normal tissues and organs, SMP30 protein levels have been evaluated using immunohistochemical (IHC) staining. As shown in Fig. 1, there is a statistically significant higher expression of SMP30 in liver tissue compared with other tissues, such as lung, spleen, myocardium, prostate and skin (*P* < 0.05).

### The expressions of SMP30 mRNA in HCC tissues

**Figure 2:**
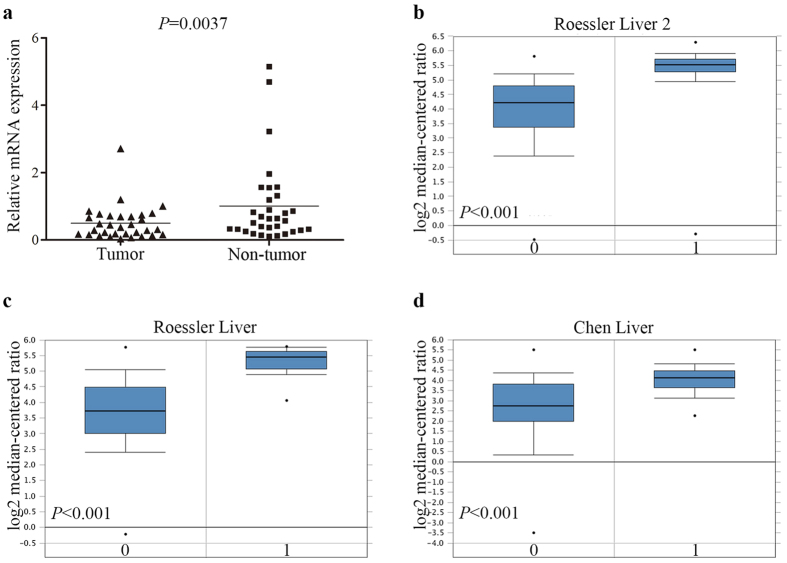
Showing expressions of SMP30 mRNA decreasing in HCC. (**a**) Relative mRNA expression of SMP30 in 32 paired HCC and adjacent tissues detected by qPCR. Oncomine data mining analysis of SMP30 mRNA levels in (**b**) Roessler Liver2, (**c**) Roessler Liver and (**d**) Chen Liver grouped by HCC (0) and normal liver (1).

In order to compare SMP30 expression levels in paired HCC and its adjacent tissues, the SMP30 mRNA expression has been evaluated using real-time quantitative PCR. We have detected significant down-regulations of SMP30 expression in 32 matched tumors as well as their adjacent non-tumor tissues, as shown in Fig. 2a. Furthermore, three independent microarray datasets from publicly available Oncomine databases^7^,^8^ have been analyzed. Figure 2b to 2d have shown that mRNA expression levels of SMP30 are consistent in our experiment data.

### The SMP30 protein expression in HCC

**Figure 3:**
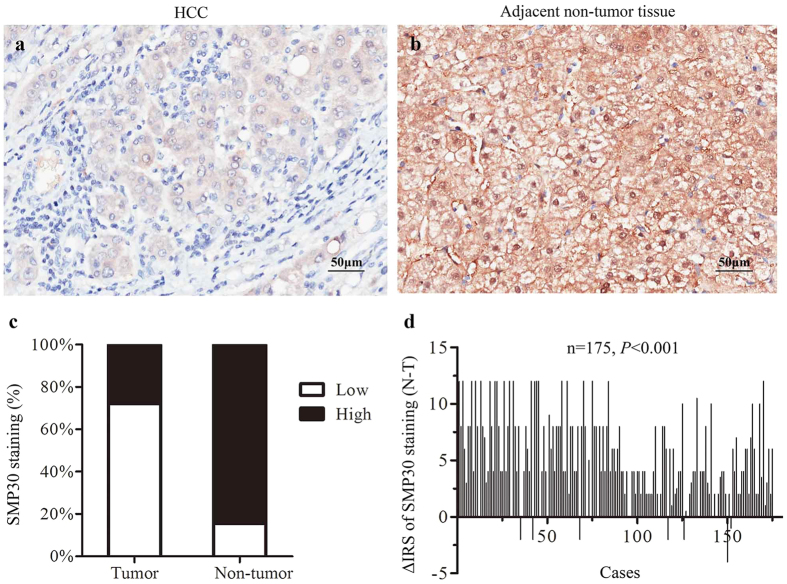
Showing the expressions of SMP30 protein in tumor and in paired adjacent non-tumor tissues. (**a**,**b**) Immunohistochemical representative images of SMP30 expression in matched tumors and in adjacent non-tumor tissues. (**c**) SMP30 expression staining is lower in HCC tissues than those in paired adjacent non-tumor tissues. Immunohistochemical staining data were from 175 pairs of tissues. (**d**) The distribution of the difference in SMP30 staining (ΔIRS = IRSN-IRST). IRS was from 175 pairs of tissues (Note: T: tumor tissues; N: non-tumor tissues). The *P* values were obtained by the Wilcoxon method.

In order to evaluate SMP30 protein expression in HCC, we have performed an immunohistochemistry analysis of SMP30 expression using tissue microarrays (TMA) containing 175 paired HCC samples (Fig. 3a and b). Samples with immunoreactive score (IRS) 0–3 are classified as low expressions and samples with IRS 4–12 as high expressions. The SMP30 low expression staining has been observed in 126 of 175 (72%) HCC tissues and 27 of 175 (15%) non-tumor tissues (Fig. 3c). These results indicate that the expressions of SMP30 in HCC tissues are significantly lower than those in paired adjacent non-tumor tissues (*P* < 0.001) as shown in Fig. 3d.

### Decreased SMP30 expression correlates with clinicopathological parameters in HCC patients

Pearson Chi-square test has been employed to study the association between SMP30 protein expression and clinicopathological properties, which include gender, age, liver cirrhosis, size, histopathologic grading, capsular formation, vascular invasion and TNM. We have discovered that the decreased expression of SMP30 is noticeably related to larger tumor sizes (*P* = 0.012) and advanced TNM stage (*P* = 0.009). There are no significant differences among SMP30 expression and gender, age, liver cirrhosis, histopathologic grading, capsular formation or vascular invasion as shown in Table 1.

### Relationship between SMP30 expression and prognosis in HCC patients

**Figure 4:**
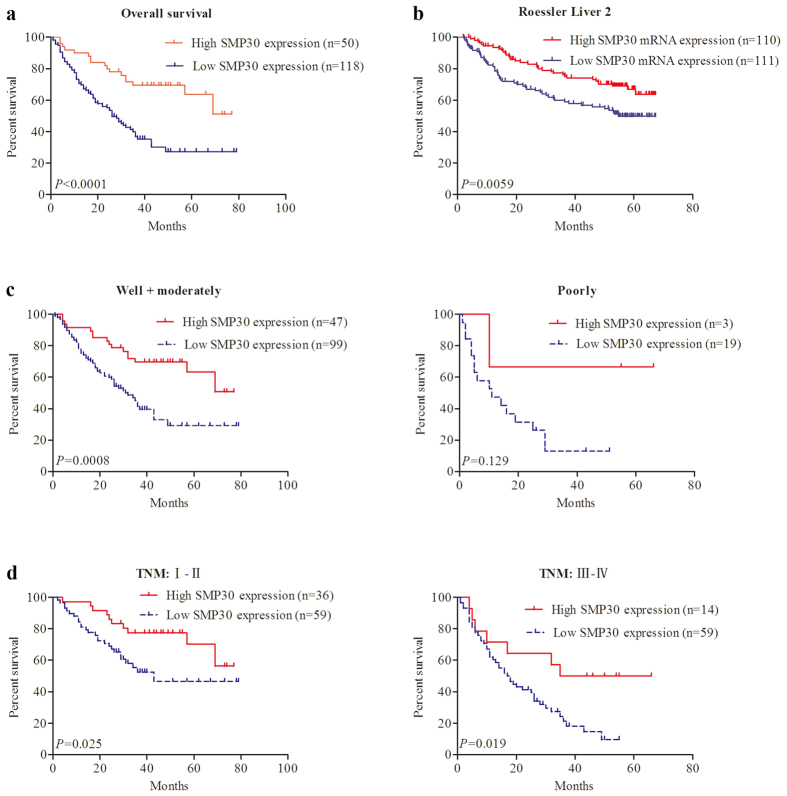
Showing SMP30 expression associated with OS rate in HCC patients. (**a**) Kaplan-Meier curves for OS in relation to SMP30 staining for 168 HCC patients. (**b**) Kaplan-Meier survival curve analysis of HCC patients with SMP30 mRNA higher-than-median or lower-than-median using Roessler Liver 2 dataset from Oncomine database. (**c**) Kaplan-Meier curves for OS in relation to SMP30 expression in well and moderately differentiated cohort and in poor differentiated cohort. (**d**) Kaplan-Meier curves for OS in relation to SMP30 expression in TNM stage (I–II) cohort and in TNM stage (III–IV) cohort. *P* values have been obtained using log-rank method.

In order to investigate whether reduced SMP30 staining in HCC patients correlates to worsening prognosis, Kaplan-Meier survival curves have been constructed using overall 7-year survived patients to compare the difference between high and low SMP30 staining value (n = 168, follow-up time, 84 months). As shown in Fig. 4a, low SMP30 expression is related to poor OS. (*P* < 0.001).

Moreover, we have performed data mining and analyses of HCC patients with different SMP30 mRNA levels from publicly available Oncomine databases to confirm the prognosis significance of SMP30 in HCC. Low levels of SMP30 are found to be related to poor OS rate of HCC patients, as revealed by the Roessler Liver 2 dataset (*P* = 0.0059)^8^ (Fig. 4b), suggesting that down-regulated SMP30 gene expression predicted poor prognosis of HCC patients.

Furthermore, SMP30 expression and its correlations with OS in HCC patients have also been evaluated using histopathologic grading and TNM stage. The analyses by Kaplan-Meier method have implied that shorter survival times in HCC patients are associated with reduced expression of SMP30 independent of histopathologic grading (Fig. 4c) and TNM stage (Fig. 4d). We have used SMP30 as an independent predictor of poor prognosis by univariate and multivariate analyses in 168 cases of HCC. The analysis using Univariate Cox regression has revealed that SMP30 expression, histopathologic grading, vascular invasion and TNM are significantly related to overall survival (Table 2).

### Genetic alterations and methylation status of SMP30

**Figure 5:**
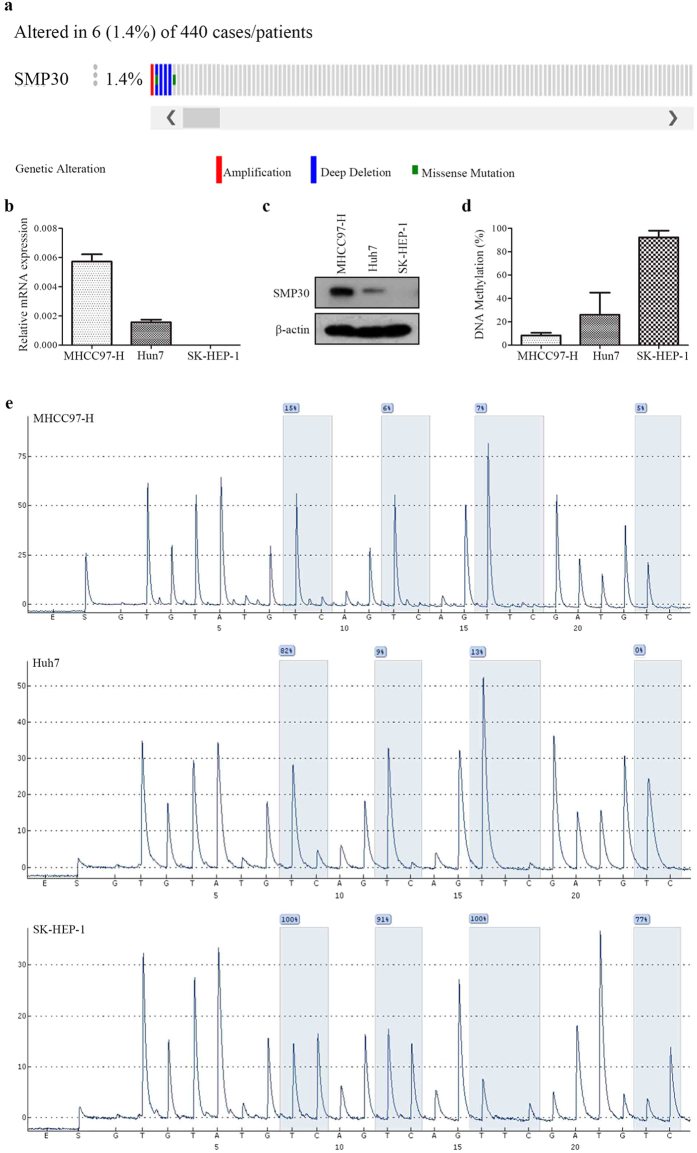
Showing genetic alterations and relationships between methylation status of SMP30 and SMP30 expression. (**a**) Genetic alteration of SMP30 provided by the cBioPortal. (**b**) Relative mRNA expression of SMP30 in HCC cell lines MHCC97-H, Huh7 and SK-HEP-1 detected by qPCR. (**c**) SMP30 protein expression in HCC cell lines MHCC97-H, Huh7 and SK-HEP-1 detected by western blotting. (**d**) The average DNA methylation levels of 4 CpG sites of SMP30 gene detected in HCC cell lines MHCC97-H, Huh7 and SK-HEP-1. (**e**) The methylation percentages of 4 CpG sites of SMP30 detected in HCC cell lines MHCC97-H, Huh7 and SK-HEP-1 by pyrosequencing.

In order to determine the frequency of genetic alterations in SMP30, we have analyzed mutation and copy number alteration data from TCGA liver hepatocellular carcinoma dataset from the cBioPortal. In the analysis depicted in Fig. 5a, each column represents a tumor sample. In total, genetic alterations of SMP30 have been identified in 6/440 (1.4%), with 1 in amplification, 2 in missense mutation, and 4 in deep deletion. The very low frequency of genetic alterations in SMP30 indicates that other mechanisms, such as epigenetic changes, accounts for SMP30 loss in HCC patients.

The DNA methylation status of 4 CpG sites of SMP30 has been evaluated by pyrosequencing (Fig. 5e). HCC cell lines MHCC97-H, Huh7 and SK-HEP-1 have shown a gradual decrease in SMP30 mRNA as well as protein levels (Fig. 5b and c), while a gradual increase in the average DNA methylation levels of 4 CpG sites has been observed (Fig. 5d). It is possible that DNA methylation mediated SMP30 gene silencing in HCC.

### Biological pathway enrichment and biological process annotation

**Figure 6:**
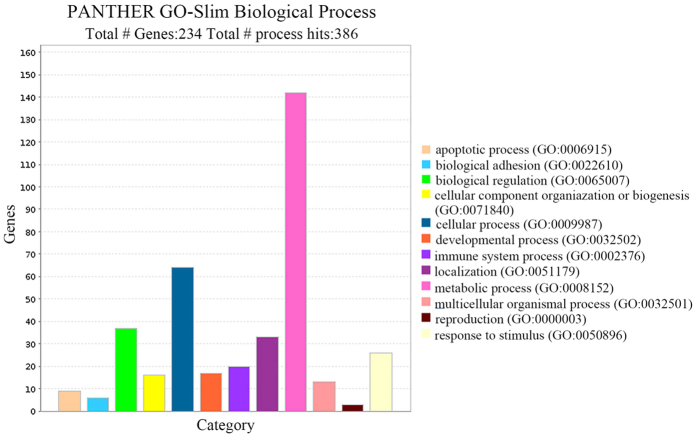
Showing biological process annotation. Bar charts display the 12 biologic processes of genes co-expressed with SMP30.

In order to reveal the biological significance of SMP30 in HCC, we have used the TCGA data for HCC (TCGA, Provisional) to screen genes co-expressed with SMP30 using the cBioPortal. Biological pathway enrichment and biological process annotation have been performed on the genes co-expressed with SMP30 using the PANTHER. The PANTHER pathway enrichment analyses have identified blood coagulation pathway, whereas a total of 12 biological processes have been identified. The genes co-expressed with SMP30 have been annotated to various processes as detailed in Fig. 6.

## Discussion

It has been reported that SMP30 is a tumor-related molecule of HCC^9^. As we were not sure whether it is commonly expressed in human normal tissues and organs, the SMP30 expression has been evaluated using immunohistochemical staining. The preferentially expression of SMP30 in normal liver has been observed. The tissue-specific pattern of protein expression can indicate important clues about gene functionalities and it has been indicated that SMP30 plays an important role in human normal liver tissue.

Previous work has shown that SMP30 can be a useful serum marker for screening and monitoring HCC patients^10^. The investigations of SMP30 expression status associated with the clinicopathological features and prognostic significance in HCC tissues, however, have not been reported. In this study, we have concluded that SMP30 expression decreases in HCC patients when comparing with that in adjacent non-tumor tissues at mRNA as well as protein levels. We have also discovered that lower SMP30 staining significantly associates with larger tumor size and enhanced TNM in HCC patients. The HCC has poor survival rate and high postoperative recurrence rate^11^. Previous studies have suggested that SMP30 has putative functions in cell proliferation, cell cycle, apoptosis and invasion^6^. It has also been concluded that high SMP30 expression suppresses proliferation in cloned rat hepatoma H4-II-E cells^12^. Our results suggest that low SMP30 expression is related to poor OS. Similar results have been confirmed from non-overlapping data in Oncomine database. Furthermore, univariate Cox regression analyses indicate that SMP30 expression, histopathologic grading, vascular invasion and TNM are significantly related to OS. These findings have implied that SMP30 could be involved in the progression of HCC and it may be an important predictor of poor prognosis in studying HCC patients.

We have used the Oncoprint feature of the cBioPortal to determine the mutation and copy number alteration frequency of SMP30 in HCC, and the SMP30 has been found less altered in 6 (1.4%) of queried samples. Instead, the methylation modification of SMP30 gene has been observed in three HCC cell lines by pyrosequencing. Our results indicate that lower CpG methylation appear in the SMP30 high-expression HCC cell line MHCC97-H and higher CpG methylation appear in the SMP30 low-expression HCC cell line SK-HEP-1. The DNA methylation-mediated transcriptional silencing has been implicated as a cause of liver cancer^13^,^14^. It seems that SMP30 genetic alterations are not the main mechanism that accounts for SMP30 loss in HCC patients and the effect of methylation modification on SMP30 expression need to be investigated further.

The PANTHER-based analyses have identified a total of 12 biological processes related to the 234 genes co-expressed with SMP30. Among the 12 biological processes, apoptotic process^15^,^16^ and biological adhesion^17^,^18^ are related to the occurrence, development and metastasis of tumor. Previous studies^19^ have demonstrated that overexpression of SMP30 suppresses the expression of oncogenes c-myc and Haras, but encourages the expression of tumor suppressor gene p53 in rat hepatoma cell line H4-II-E. The inhibition of SMP30 in HCC cell line Hep G2 has been shown to promote the apoptosis and invasion^6^. PANTHER pathway enrichment analysis identified blood coagulation pathway. It has been widely recognized that blood coagulation is correlated to cancer development and thrombin contributes to tumor metastasis via increasing the adhesive potential of malignant cells^20^,^21^. We tentatively put forward that genes co-expressed with SMP30 may affect the prognosis through participating in processes such as apoptotic process, biological adhesion, and blood coagulation.

In summary, we believe that the low SMP30 expression is strongly correlated to poor OS rate of HCC patients and the findings may be useful in developing a reliable prognostic marker and a potential HCC therapeutic target.

## Methods

### Patients and specimens

Human normal tissue microarray (TMA) and two independent HCC cohorts TMA were purchased from Shanghai Outdo Biotech, China. Human normal TMA (catalog no. HOrg-N090-01) included 24 organs from 2–7 normal persons. HCC TMA (catalog no. HLiv-HCC180Sur-05) included 95 HCC tissues and 85 paired adjacent non-tumor tissues, HCC TMA (catalog no. HLiv-HCC180Sur-03) included 90 HCC tissues and the same amount of paired adjacent non-tumor tissues. The operations were carried out within August 2006 to September 2011, the last follow-up time was September 2013. Follow-up information was missing for 17 cases. An additional 32 paired formalin-fixed paraffin-embedded HCC tissues and corresponding adjacent non-tumor tissues were obtained from the affiliated hospital of Guangxi Medical University, China. Informed consent was obtained from all patients for the use of tissues in experimental procedures. This study was approved by the Ethics Committee of Guangxi Medical University. All methods were performed in accordance with Guangxi Medical University guidelines and regulations.

### Cell culture

Human HCC cell lines SK-HEP-1 and Huh7 were purchased from the Cell Bank of Chinese Academy of Sciences, Kunming. HCC cell line MHCC97-H was purchased from FuDan IBS Cell Center (FDCC), Shanghai. Cells were cultured in 5% CO2 at 37 °C with high glucose Dulbecco’s modified Eagle media (Gibco, USA) supplemented with penicillin (100 IU/mL), streptomycin (100 mg/mL) and 10% FBS (Gibco, USA).

### IHC and evaluation of immunostaining

Immunohistochemistry was performed with the SMP30 antibody at a dilution of 1:6000 according to a commercial protocol of Shanghai Outdo Biotech. Antibody staining was visualized with DAB and hematoxylin counterstain. The staining intensities of SMP30 were scored from 0 to 3 where 0 means negative, 1 weak, 2 moderate and 3 strong. The percentages of positively stained cells were scored in scales of 0 to 4, where 1 (0–25%), 2 (26–50%), 3 (51–75%) and 4 (76–100%). The scores for percentages of positive cells and staining intensities were then multiplied to generate an IRS for each case. The IRS ranged from 0–12. Cut-off levels for this scoring system were assigned as follows: high SMP30 expression was defined as an IRS of ≥ 4; and low SMP30 expression was defined as an IRS of < 4^22^. These scores were assessed by 2 pathologists simultaneously.

### The RNA isolation and quantitative real-time PCR

The E.Z.N.A. Total RNA Kit (Omega, USA) was used to isolate total RNA for cell lines according to instructions from manufacturer. For formalin-fixed paraffin-embedded samples, total RNA was isolated using E.Z.N.A. FFPE RNA Isolation Kit (Omega, USA) according to instructions from manufacturer. The cDNA was obtained by employing a PrimeScript RT-PCR kit (Takara, Japan) following the instructions of the manufacturer. The qPCR with SYBR green I was employed for comparing relative expression of specific mRNAs and the procedures have been described elsewhere^23^. The expression level of mRNA was normalized to GAPDH. The primer sequences for SMP30 and GAPDH are shown as following: SMP30: 5′-GTGGATGCCTTTGACTATGACC-3′ (forward), 5′-CTTCCCCTCAGCATCAATACAC-3′ (reverse). GAPDH: 5′-TGCACCACCAACTGCTTAGC-3′ (forward), 5′-GGCATGGACTG TGGTCATGAG-3′ (reverse). The running conditions were as follows: 95 °C for 2 min, followed by 40 cycles of 95 °C for 15 sec, 55 °C for 30 sec and 72 °C for 30 sec. All samples were run in triplicate in 20 μl final volume reaction containing 2 μl diluted cDNA, 20pmol/ul primers, 10 μl SYBR Premix Ex Taq™ and 0.4 μl ROX. The relative expression of SMP30 to GAPDH was computed from the delta Ct method.

### Western blotting

Protein extracts from the HCC cell lines and Western blots were performed as described^24^. Equal amounts of proteins from HCC cell lines were subjected to western blot. Antibodies used were anti-SMP30 antibodies and anti-β-actin antibody (Abcam, UK).

### Pyrosequencing analysis of SMP30

Genomic DNA was isolated using the AxyPrep™ Genomic DNA Miniprep Kit (Qiagen, Germany). Genomic DNA was bisulfite converted using the EpiTect Bisulfite Kit (Qiagen, Germany) according to instructions from manufacturer. The PyroMark™ software (Qiagen, Germany) was used to design pyrosequencing primers. Forward primer: 5′-GGGGAGGGGTTTTAGGTTTATGT-3′, reverse primer: 5′-ATCTCCTTCTAACAATCAACTATCA-3′, sequencing primer: 5′-GGGTTTTAGGTTTATGTTATTTAT-3′. PCR reactions were as follows: 95 °C for 3 min, followed by 40 cycles of 94 °C for 30 sec, 54 °C for 30 sec and 72 °C for 1 min, followed by a final extension of 7 min at 72 °C. The size of the amplified product containing 4 CpG sites was 246 bp. The specific PCR products were sequenced following the manufacturer’s protocol using a PyroMark Q96 ID (Qiagen, Germany).

### Bioinformatics analyses

We analyzed the mRNA expression level of identified proteins in HCC using Oncomine, which is a cancer microarray database and integrated data-mining platform accessible from https://www.oncomine.org/resource/login.html 25,26. We subsequently performed alteration analysis and gene screening on The Cancer Genome Atlas (TCGA) data for HCC (TCGA, Provisional) by using cBioPortal (http://www.cbioportal.org/)^27^. The cBioPortal data were subjected to scheduled updates. Biologic process and pathway analysis of genes co-expressed with SMP30 was then performed using the PANTHER (http://pantherdb.org)^28^.

### Statistical analysis

Student’s t test was employed for analyzing quantitative variables. The association between SMP30 staining and the clinicopathologic parameters of the HCC patients was evaluated by the Pearson chi-square test. Wilcoxon test (grouped) was used to estimate differences in IRS for SMP30 staining in primary tumors and in their paired adjacent non-tumor tissues. Survival curves were evaluated using Kaplan-Meier method and the differences between those survival curves were tested by log-rank test. The analyses of univariate and multivariate were based on the Cox model using SPSS 20.0. It was considered statistically when a two-sided P-value is less than 0.05.

## Additional Information

**How to cite this article**: Mo, Z. *et al*. Senescence marker protein 30 (SMP30) serves as a potential prognostic indicator in hepatocellular carcinoma. *Sci. Rep.* **6**, 39376; doi: 10.1038/srep39376 (2016).

**Publisher's note:** Springer Nature remains neutral with regard to jurisdictional claims in published maps and institutional affiliations.

## Figures and Tables

**Table 1:** Clinicopathological correlation of SMP30 expression in 168 HCC patients using Pearson’s x^2^ test.

Clinicopathological feature	Expression of SMP30		
	High	Low	*P* Value
Gender			0.168
Male	40	104	
Female	10	14	
Age (years)			0.484
≤ 50	15	42	
> 50	35	76	
Liver cirrhosis			0.099
Absent	30	86	
Present	20	32	
Size (cm)			**0.012**
≤5	30	46	
>5	20	72	
Histopathologic grading			0.076
Well + moderately	47	99	
Poorly	3	19	
Capsular formation			0.203
Absent	4	18	
Present	46	100	
Vessel invasion			0.934
Absent	46	109	
Present	4	9	
TNM			**0.009**
I–II	36	59	
III–IV	14	59	

Bolded *P* values represent statistical significance.

**Table 2:** Univariate and multivariate analyses of factors associated with OS, where HR is Hazard Ratio and CI Confidence Interval.

Prognostic paramerer	Univariate analysis			Multivariate analysis		
	HR	95%CI	*P* value	HR	95%CI	*P* value
Expression of SMP30(low vs. high)	2.656	1.532–4.605	**0.001**	2.681	1.554–4.627	**0.000**
Gender (female vs. male)	0.578	0.276–1.207	0.144	—	—	—
Age (≤50 vs. >50)	1.138	0.728–1.778	0.570	—	—	—
Liver cirrhosis (present vs. absent)	1.283	0.792–2.079	0.311	—	—	—
Size (≤5 vs. >5)	0.945	0.526–1.698	0.851	—	—	—
Histopathologic grading (Well/moderately vs. Poorly)	0.513	0.291–0.903	**0.021**	0.559	0.324–0.967	**0.037**
Capsular formation (present vs. absent)	1.200	0.639–2.253	0.570	—	—	—
Vessel invasion (present vs. absent)	2.141	1.030–4.447	**0.041**	2.488	1.283–4.821	**0.007**
TNM (I/II vs. III/IV)	0.447	0.248–0.804	**0.007**	0.460	0.294–0.719	**0.001**

The *P* values in bold represent statistical significance.
